# Frequent mismatch-repair defects link prostate cancer to Lynch syndrome

**DOI:** 10.1186/s12894-016-0130-1

**Published:** 2016-03-24

**Authors:** Mev Dominguez-Valentin, Patrick Joost, Christina Therkildsen, Mats Jonsson, Eva Rambech, Mef Nilbert

**Affiliations:** Institute of Clinical Sciences, Division of Oncology and Pathology, Lund University, SE-22381 Lund, Sweden; HNPCC-Register, Clinical Research Centre, Copenhagen University Hospital, Hvidovre, Denmark

**Keywords:** Mismatch repair deficiency, Microsatellite instability, *MLH1*, *MSH2*, *MSH6*

## Abstract

**Background:**

A possible role for prostate cancer in Lynch syndrome has been debated based on observations of mismatch-repair defective tumors and reports of an increased risk of prostate cancer in mutation carriers. Potential inclusion of prostate cancer in the Lynch syndrome tumor spectrum is relevant for family classification, risk estimates and surveillance recommendations in mutation carriers.

**Methods:**

We used the population-based Danish HNPCC-register to identify all prostate cancers that developed in mutation carriers and in their first-degree relatives from 288 Lynch syndrome families. The tumors were evaluated for clinicopathologic features and mismatch-repair status, and the cumulative risk of prostate cancer was determined.

**Results:**

In total, 28 prostate cancers developed in 16 mutation carriers and in 12 first-degree relatives at a median age of 63 years. The majority of the tumors were high-grade tumors with Gleason scores 8–10. Prostate cancer was associated with mutations in *MSH2, MLH1* and *MSH6* with loss of the respective mismatch repair protein in 69 % of the tumors, though a MSI-high phenotype was restricted to 13 % of the tumors. The cumulative risk of prostate cancer at age 70 was 3.7 % (95 % CI: 2.3–4.9).

**Conclusion:**

We provide evidence to link prostate cancer to Lynch syndrome through demonstration of MMR defective tumors and an increased risk of the disease, which suggests that prostate cancer should be considered in the diagnostic work-up of Lynch syndrome.

## Background

Lynch syndrome is a multi-tumor syndrome with the highest risks for colorectal cancer and endometrial cancer though a number of other tumor types, e.g. cancer of the urinary tract, the small bowel and the ventricle, ovarian cancer, brain tumors and skin tumors develop at increased incidence [1, 2]. Other tumor types assumed to represent sporadic tumors in families with hereditary cancer, e.g. breast cancer, pancreatic cancer and sarcoma may indeed develop as part of the syndrome. This is suggested based on identification of mismatch repair (MMR) defective tumors of these subtypes and demonstration of an increased risk of these tumor types in mutation carriers [3–10].

Prostate cancer is the most common tumor type in men in the Western world with an estimated lifetime risk of 18 % and a median age at diagnosis of 67 years [1]. Worldwide, prostate cancer is the sixth common tumor with more than 250,000 deaths annually [11]. In Denmark, prostate cancer constitutes 23 % of all male cancers with an estimated risk of 10 % for disease development before age 75 [12]. The role of prostate cancer in Lynch syndrome is unresolved though molecular investigations and epidemiologic studies have suggested a potential link to the syndrome [1, 8, 13]. The MMR system has been suggested to influence prostate carcinogenesis e.g. through an increased risk of prostate cancer linked to single nucleotide polymorphisms in *MLH1* and *MSH3,* and a role for complex structural rearrangements in *MSH2* and *MSH6* as a mechanism underlying the hypermutation in aggressive prostate cancer [14–20]. We assessed the role of prostate cancer in the Danish Lynch syndrome cohort with characterization of MMR status and risk estimates.

## Methods

### Patients and tumor samples

The Danish Hereditary Non-Polyposis Colorectal Cancer (HNPCC) Register is a national Danish register containing all families identified with proven or suspected hereditary cancer. Through research collaborations, data from the register is freely available. We obtained data on all adenocarcinomas of the prostate that had developed in carriers of a disease-predisposing MMR gene mutation in *MLH1, MSH2, MSH6* or *PMS2* and in their first-degree relatives. Clinical data were obtained from pathology reports and clinical files. All patients provided an informed consent for inclusion into the Danish HNPCC register during genetic counseling sessions. Ethical approval for the study was granted from the Ethical Committee at The Capital Region of Copenhagen, Denmark (H-D-2007–0032). All tumor specimens available were collected for analysis of MMR status. The tumors were pathologically reviewed regarding their Gleason scores and the presence of tumor-infiltrating lymphocytes (TIL) (cut-off ≥4 per high-power field) [8, 21] by a pathologist (PJ), who was blinded to MMR status.

### Immunohistochemistry and analysis of microsatellite instability

All tumors were immunohistochemically stained for the MMR proteins MLH1, PMS2, MSH2 and MSH6. Briefly, 4-μm sections were placed on SuperFrost® Plus microscope slides. Antigen retrieval was performed in a pressure boiler in Target Retrieval Solution, pH 9 (Dako, Glostrup, Denmark) and stained in an automated immunostainer (Autostainer Plus, Dako, Glostrup, Denmark) using Dako EnVision™FLEX+ Detection System, Peroxidase/DAB, Rabbit/Mouse (Dako, Glostrup, Denmark), according to the manufacturers' instructions. The antibodies used were MLH1, clone ES05 (Dako, Glostrup, Denmark, dilution 1:100), PMS2, clone A16-4 (BD Pharmingen, San Diego, CA, dilution 1:300), MSH2, clone FE11 (Calbiochem, Merck KgaA, Darmstadt, Germany, dilution 1:100), and MSH6, clone EPR3945 (Epitomics, Burlingame, dilution 1:100). Tumor MMR protein expression was assessed as retained (normal), lost, or reduced (i.e. tumor cell staining intensity was reduced compared with that of the normal internal control).

For analysis of microsatellite instability (MSI), non-necrotic tumor areas were macro-dissected from the paraffin-embedded tumor blocks. DNA extraction was performed from three 5-mm sections using the Qiagen FFPE Kit (Qiagen Valencia, CA) according to the manufacturer’s instructions. DNA concentration was determined using a Qubit Fluorometric Quantitation (Invitrogen) and the products run on a 3130XL Genetic Analyzer (Applied Biosystems, Foster City, CA). The analysis was performed using the MSI Analysis System, Version 1.2 (Promega, Madison, WI) and included the 5 mononucleotide markers BAT-25 BAT-26, NR-21, NR-24, and MONO-27 (Promega, MSI Analysis System, Version 1.2, Madison, WI). The results were evaluated using GeneMapper Software Version 4.0 (Applied Biosystems, Foster City, CA) and defined as MSI high when ≥2 markers were unstable, MSI low when one marker was unstable and MSS when none of the markers were unstable.

### Statistical analysis

Genotypic and phenotypic data from all mutation carriers and their first-degree relatives were transferred into R i386 3.1.0 (R: A Language and Environment for Statistical Computing, 2011, R Foundation for Statistical Computing, Vienna, Austria). *EPCAM* mutations (identified in one family) were pooled together with *MSH2* mutations, while 7 *PMS2* mutation families (none of which contained any prostate cancers) were excluded from the analyses. Mutation carriers were weighted by 1 and first-degree relatives by 0.5 motivated by a 50 % risk of carrying the inherited mutation. The event times used were age at diagnosis, age at death or current age (censored at May 14, 2014). Cumulative incidences were calculated with death as a competing risk (cmprsk: Subdistribution Analysis of Competing Risks, 2011, Bob Gray, R package version 2.2-2). Confidence intervals were calculated at age 70 using a non-parametric bootstrap. Permutation tests with 10,000 replicates were used to calculate *p*-values with significance set at *p* < 0.05.

## Results

In total, 288 Lynch syndrome families with disease-predisposing germline mutations in *MLH1, MSH2, MSH6* or *PMS2* were identified in the Danish HNPCC register. In this cohort of 1609 males (677 mutation carriers and 932 first-degree relatives), prostate cancer developed in 16 mutation carriers and in 12 first-degree relatives. The median age at diagnosis of prostate cancer was 61 (range 52–78) years for the mutation carriers and 63 (range 53–81) years for the first-degree relatives. All tumors were adenocarcinomas with Gleason scores between 6 and 10. The tumors were linked to disease-predisposing mutations in *MSH2* (*n* = 14), *MLH1* (*n* = 8) and *MSH6* (*n* = 6) (Table 1). Among the 28 men diagnosed with prostate cancer, 16 had a previous cancer diagnosis, which included colon cancer in 15 cases. Four prostate cancers had developed among the 593 male non-mutation carriers (0.67 %) compared to 2.22 % of the mutation carriers and 1.39 % of the first-degree relatives.

**Table 1:** Prostate cancers analyzed for mismatch-repair function

ID no.	Status	Age at diagnosis	Gleason score	TILs ≥4/HPF	MMR gene mutation	Immunohistochemical staining				MSI
						MLH1	PMS2	MSH2	MSH6	
P32	Carrier	56	9 (4 + 5)	NA	*MSH2 c.(?_-68)_366 + ?del*			NA		
P7	Carrier	53	8 (4 + 4)	y	*MSH2* c.560G > T	+	+	−	−	MSI-L
P14	FDR	63	9 (5 + 4)	y	*MSH2* c.646-?_1276 + ?del	+	+	−	−	MSI-L
P8	Carrier	69	9 (4 + 5)	y	*MSH2* c.892C > T	+	+	−	−	MSI-L
P33	FDR	77	NA	NA	*MSH2 c. 942 + 3A > T*			NA		
P34	FDR	62	NA	NA	*MSH2 c. 942 + 3A > T*			NA		
P5	Carrier	69	8 (3 + 5)	NA	*MSH2 c. 942 + 3A > T*			NA		
P31	FDR	56	10 (5 + 5)	NA	*MSH2 c. 942 + 3A > T*			NA		
P10	Carrier	76	7 (4 + 3)	y	*MSH2* c.1786_788delAAT	+	+	−	−	MSI-L
P20	FDR	81	10 (5 + 5)	y	*MSH2* c.1786_788delAAT	+	+	−	−	MSI-H
P9	Carrier	52	8 (4 + 4)	y	*MSH2* c.1906G > C	+	+	−	−	MSS
P30	Carrier	60	NA	NA	*MSH2 c.2038C > T*			NA		
P12	Carrier	57	6 (3 + 3)	y	*MSH2* c.2347delC	+	+	−	−	MSI-H
P35	Carrier	67	NA	NA	*MLH1 c. 350C > T*			NA		
P11	Carrie	63	7 (4 + 3)	n	*MLH1* c.588 + 5G > A	+	+	+	+	MSS
P22	FDR	56	7 (3 + 4)	y	*MLH1* c.1537_1547delInsC	−	−	+	+	MSS
P2	Carrier	60	8 (4 + 4)	n	*MLH1* c.1667 + 2delTAAATCAinsATTT	+	+	+	+	MSS
P13	FDR	63	9 (4 + 5)	y	*MLH1* c.1667 + 2delTAAATCAinsATTT	−	−	+	+	MSI-L
P18	FDR	63	7 (3 + 4)	y	*MLH1* c.1667 + 2delTAAATCAinsATTT	+	+	+	+	MSS
P6	FDR	80	7 (3 + 4)	n	*MLH1* c.1732-2A > T	+	+	+	+	MSS
P1	Carrier	72	NA	NA	*MLH1 c.1732-2A > T*			NA		
P16	Carrier	74	10 (5 + 5)	n	*MLH1* c.1852_54delAAG	+	+	+	+	MSS
P37	Carrier	58	NA	NA	*MSH6 C.1444C > T*			NA		
P3	Carrier	58	8 (4 + 4)	y	*MSH6* c.1483C > T	+	+	−	−	MSI-L
P15	Carrier	78	6 (3 + 3)	y	*MSH6* c.3647-1G > A	+	+	+	−	MSS
P38	FDR	81	NA	NA	*MSH6 c. 3609_3612delTGCA*			NA		
P36	FDR	53	NA	NA	*MSH6 c.3992 + 1 T > C*			NA		
P17	FDR	57	7 (3 + 4)	NA	*MSH6 c.3992 + 1 T > C*			NA		

*Abbreviations*: *FDR* first-degree relatives, *HPF* high-power field, *MMR* mismatch repair, *MSI* microsatellite instability high/low, *MSS* microsatellite stability, *n* no, *TIL* tumour-infiltrating lymphocytes, *y* yes, *NA* Not available

**Fig. 1:**
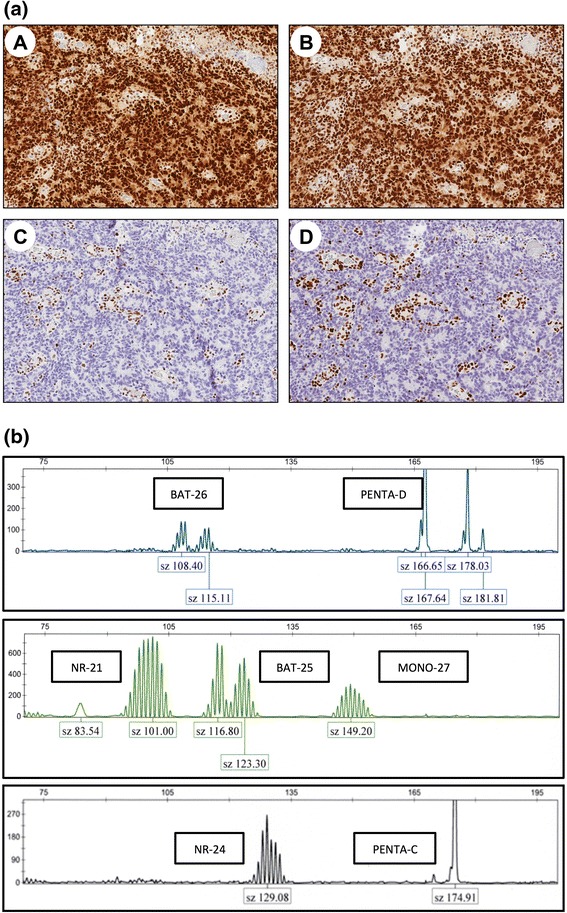
**a** A prostate cancer from an individual with a *MSH2* mutation showing normal expression for MLH1 and PMS2 (**A** and **B**) and loss of expression for MSH2 and MSH6 (**C** and **D**); **b** microsatellite instability for the markers BAT-26, NR-21, BAT-25, NR-24 and MONO-27 in the same prostate cancer

Tumor tissue could be retrieved for MMR analysis from 16 tumors (derived from 10 mutation carriers and 6 first-degree relatives), with loss of expression for the respective MMR proteins in 11/16 tumors (including 7/10 tumors from mutation carriers (Table 1). Notably, MMR protein loss was detected in all *MSH2* and *MSH6* associated tumors prostate cancers. MSI analysis applied standard diagnostic markers and revealed a MSI-high phenotype in 2 tumors, a MSI-low phenotype in 6 tumors and a MSS phenotype in 8 tumors (Table 1, Fig. 1). Notably, all *MLH1*-associated tumors has a microsatellite stable phenotype. Pathologic review revealed TIL in 12/16 tumors, including all MMR defective tumors. Gleason scores tended to be high in MMR defective prostate cancer with Gleason scores of 8–10 in 7/11 MMR defective prostate cancers (Table 1).

**Fig. 2:**
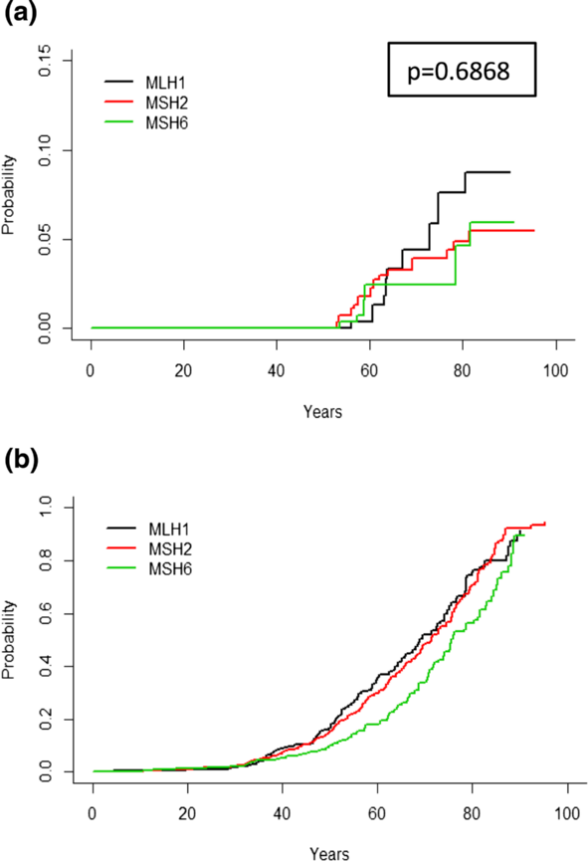
Non-parametric risk estimates showing **a** age-specific cumulative risks for prostate cancer; **b** mortality rates in *MLH1*, *MSH2* and *MSH6* families

Risk analysis could be performed based on 1488/1609 males from whom complete data were available. The cumulative risk for prostate cancers at age 70 was 3.7 % (95 % CI: 2.32–4.92) in mutation carriers and first-degree relatives compared to 593 for non-mutation carriers in these families (Fig. 2a). No significant differences could be demonstrated in relation to disease-predisposing gene; *MLH1* 4.4 % (95 % CI: 1.44; 7.04), *MSH2* 3.9 % (95 % CI: 1.96–5.70) and *MSH6* 2.5 % (95 % CI: 0.56–4.12) (Fig. 2b).

## Discussion

In the Danish Lynch syndrome cohort, 28 prostate cancers were identified. These tumors were diagnosed at a median age of 63 years, which is in line with reports of prostate cancers diagnosed at median 59–65 years in Lynch syndrome [9, 22–26]. MMR protein loss in line with the underlying MMR gene mutation was identified in 11/16 prostate cancers, including all *MSH2* and *MSH6* mutant tumors and supports observations of a high degree, 69–100 %, of MMR deficiency in prostate cancers in Lynch syndrome [8, 24, 27]. *MSH2* mutations were found in 14/28 prostate cancers and this MMR gene has been linked to an expanded spectrum of extracolonic tumors with an increased risk for e.g. urothelial cancer, brain tumors and skin tumors [6, 8–10, 22–24, 28, 29]. A MSI-high phenotypes was identified only in 2 prostate cancers using a standard MSI markers panel with an additional number of tumors showing a MSI-low phenotype. This observation supports an earlier report of MSI defects in 4–10 % of Lynch syndrome associated prostate cancers [30]. MSI phenotypes were predominantly observed in *MSH2* and *MSH6* associated tumors and could potentially reflect alternate mechanisms by which MSI is acquired in prostate cancer or an association with tumor differentiation [19] [31]. These observations support a role for MMR defects in prostate carcinogenesis and link germline MMR defects to the development of prostate cancer.

MMR-defective tumors show histopathologic characteristics that include poor differentiation and lymphocytic reactions with an increased number of TIL [8]. Blinded analysis of TIL showed a striking correlation with MMR defects with TIL in all MMR defective prostate cancer and in only 1/5 MSS and MMR proficient prostate cancers. TIL has been suggested to represent an adverse prognostic factor in prostate cancer [32–34]. Of the 11 MMR defective prostate cancers in our study, 7 had a Gleason score of ≥8 suggesting aggressive tumors. Hereditary prostate cancers, also associated with *BRCA2* mutations, have been suggested to have an accelerated tumor development an aggressive phenotype. Knowledge about prostate cancer in Lynch syndrome is scarce, but early age at onset, frequent TIL and an aggressive phenotype warrants further investigation related to a possible role for surveillance and potential therapeutic implications from e.g. immunotherapy with PD-1 inhibitors [35].

The cumulative risk of prostate cancer at age 70 was 3.7 % in mutation carriers. No significant differences were discerned in relation to disease-predisposing gene, but this analysis is based on very limited numbers. Under the assumption that MMR-defective prostate cancer signifies Lynch syndrome, mutation carriers can be estimated to be at a 2- to 3-fold increased risk of prostate cancer compared to the general population [1, 13]. Growing data suggest that MMR defects in prostate cancer may signify chromoplexy, whereby a single hit infers genetic complexity of relevance for prostate cancer initiation, progression and therapeutics [18, 19].

## Conclusions

The Danish Lynch syndrome cohort contains 28 prostate cancers that developed at a median age of 63 years, showed high Gleason scores and frequent TILs. The tumors were predominantly linked to *MSH2* mutations. Frequent MMR defects consistent with the underlying germline defects suggest that prostate cancer is included in Lynch syndrome tumor spectrum and should be considered during genetic counseling.

### Availability of data and materials

All available data from the cases included are summarized in Table 1. Additional data on these individuals and their families (e.g. detailed mutation data and family data) are freely available from the Danish HNPCC-register though contact with the principal investigator, Mef.Nilbert@regionh.dk.

## Abbreviations

CI: confidence interval
HNPCC: hereditary nonpolyposis colorectal cancer
MMR: mismatch repair
MSI: microsatellite instability
MSS: microsatellite stable
TIL: tumor-infiltrating lymphocytes.
